# Bridging molecular-scale interfacial science with continuum-scale models

**DOI:** 10.1038/s41467-024-49598-y

**Published:** 2024-06-22

**Authors:** Anastasia G. Ilgen, Eric Borguet, Franz M. Geiger, Julianne M. Gibbs, Vicki H. Grassian, Young-Shin Jun, Nadine Kabengi, James D. Kubicki

**Affiliations:** 1 Geochemistry Department, Sandia National Laboratories, Albuquerque, NM 87123 USA; 2 Department of Chemistry, Temple University, Philadelphia, PA 19122 USA; 3 Department of Chemistry, Northwestern University, Evanston, IL 60208 USA; 4 Department of Chemistry, University of Alberta, Edmonton, AB T6G 2G2 Canada; 5 Department of Chemistry and Biochemistry, University of California, La Jolla, CA 92093 USA; 6 Department of Energy, Environmental and Chemical Engineering, Washington University in St. Louis, St. Louis, MO 63130 USA; 7 Department of Geosciences, Georgia State University, Atlanta, GA 30302 USA; 8 Department of Earth, Environmental and Resource Sciences, The University of Texas at El Paso, El Paso, TX 79968 USA

**Keywords:** Environmental chemistry, Geochemistry, Computational chemistry

## Abstract

Solid–water interfaces are crucial for clean water, conventional and renewable energy, and effective nuclear waste management. However, reflecting the complexity of reactive interfaces in continuum-scale models is a challenge, leading to oversimplified representations that often fail to predict real-world behavior. This is because these models use fixed parameters derived by averaging across a wide physicochemical range observed at the molecular scale. Recent studies have revealed the stochastic nature of molecular-level surface sites that define a variety of reaction mechanisms, rates, and products even across a single surface. To bridge the molecular knowledge and predictive continuum-scale models, we propose to represent surface properties with probability distributions rather than with discrete constant values derived by averaging across a heterogeneous surface. This conceptual shift in continuum-scale modeling requires exponentially rising computational power. By incorporating our molecular-scale understanding of solid–water interfaces into continuum-scale models we can pave the way for next generation critical technologies and novel environmental solutions.

## Abstract (web summary)

Chemistry at solid-water interfaces is crucial for all aspects of human life. Here, authors propose to use a probability-based paradigm for formalizing chemical reactions at solid-water interfaces in continuum scale models.

## Introduction

Solid–water interfaces play critical roles in engineered systems^1–3^ and natural environments^4^. Communication among scientists and engineers working at molecular, microscopic, field, and global scales should be augmented via integrated collaborations that seek to add chemical insights into large-scale problems where current assumptions and approximations lead to large uncertainties in predictive models^5^. We lay out a perspective about how to establish such a collaboration that infuses molecular details into larger scale models, including often-used surface complexation (SCM) and reactive transport models (RTM). We propose the development of a new approach for incorporating the vast database of molecular knowledge into continuum-scale models by shifting the model parameterization paradigm. We suggest a conceptual shift in how surface properties are represented from the current state of using discrete values to probability distributions, allowing to reflect real heterogeneities of surfaces. Surface site acidities, charge densities, solvation energies, reaction rates, and solubility constants should be described as probability curves to reflect the interfacial complexity.

Scientists who develop detailed molecular descriptions of solid–water interfaces face a four-fold challenge: (1) interfacial chemistry evolves in complex ways as it is dynamically coupled to the compositions of both the solid and the aqueous phases yet is distinct from either; (2) the number of atoms present at the surface is dwarfed by the number of atoms that compose the bulk phases, thus complicating the deconvolution of surface analytical signals from those of the bulk; (3) real-world interfaces are inherently heterogeneous down to the micro-, nano-, and molecular-scales, making it difficult to build continuum-scale predictive models that capture this complexity and reconcile distinct surface structures with observed net reactivities; and (4) environmental processes span femtosecond to millennia timescales, not always accessible for experimental, analytical, and computational inquiries. Despite these challenges, previously obscure details of surface reactions are becoming increasingly understood. However, the current numerical tools available for translating interfacial processes into continuum-scale models that describe mm- to km-scale systems are lacking mathematical frameworks for incorporating the wealth of molecular details that have been discovered in the last few decades.

**Fig. 1:**
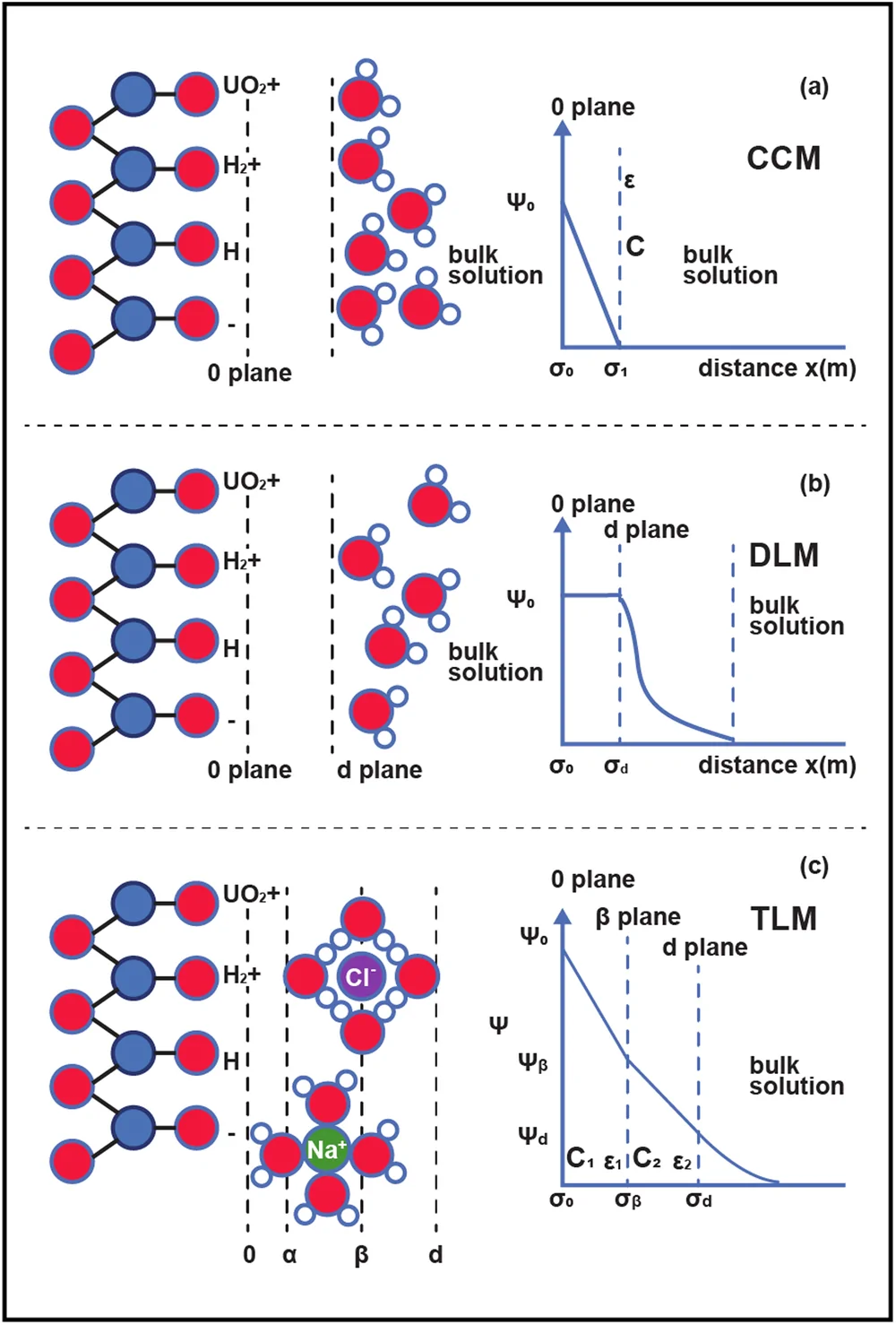
Schematic representation of surface complexation modeling. Surface complexation modeling based on **a** Constant capacitance model (CCM); **b** Diffuse layer model (DLM); and **c** Triple layer model (TLM). A *0*-plane in these models limits the solid’s surface, a *β*-plane terminates the plane where counter-ions are tightly bound at charged surfaces (Stern layer) and a *d*-plane cuts through the center of the diffuse layer near surfaces. *C*, including *C*_*1*_ and *C*_*2*_, denote individual layer capacitance values; *ψ* is the surface potential corresponding to one of the planes, and *σ*, including *σ*_*β*_ and *σ*_*d*_, is charge of the corresponding layers, where *σ*_*0*_ is charge of the *0*-plane (surface charge, or surface charge density); ε is the dielectric constant or permittivity of the media. Figure adapted with permission from ref. ^6^.

Because of these limitations, scientists who construct SCMs and RTMs often use “average” values to describe the structures and reactivities of solid–water interfaces to reflect relevant molecular information. SCMs are developed to specifically describe ion adsorption behaviors at solid–water interfaces to match either adsorption isotherms or pH-dependent adsorption data (i.e., adsorption edges). The basic schematic for three types of commonly used SCMs is shown in Fig. 1 (reproduced from ref. ^6^). These SCMs are based on various continuum-scale models of interfacial structure such as: (1) the constant capacitance model (CCM), (2) the diffuse layer model (DLM), and (3) the triple layer model (TLM). Each of these SCMs assumes that the total free energy of ion adsorption is a sum of chemical adsorption energy (ΔG_chem_) and Coulomb static energy (ΔG_coul_), where ΔG_coul_ is directly proportional to the surface potential (*ψ*) and the charge of the adsorbing ion^6^. In the sections below we illustrate that neither ΔG_chem_ nor ΔG_coul_ can be considered constants in any given interfacial system because of the variability of surface structures that define local surface charge or the reactivity of isolated surface groups, which should lead to variability in the surface potential across the same surface caused by intrinsic surface heterogeneity. Therefore, to reflect the true complexity, ΔG_chem_ and ΔG_coul_ would be best represented by a distribution of values, rather than a fixed value.

**Fig. 2:**
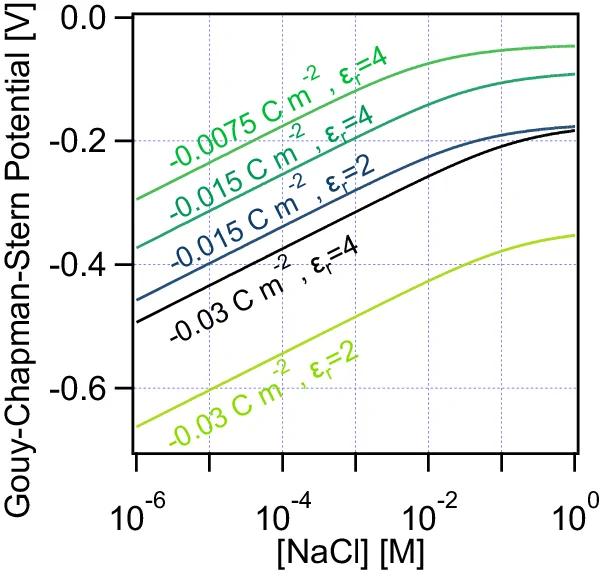
Calculated Gouy-Chapman Stern potential. Calculated variability in the ionic strength dependent Gouy-Chapman Stern (GCS) potential due to variations in charge density and Stern layer relative permittivity (ε_r_) commonly observed across surfaces. The resulting Gouy-Chapman Stern potential variations reach several hundred mV when doubling the charge density and halving the Stern layer permittivity. In contrast, doubling both parameters results in only minor potential differences.

To illustrate the sensitivity of a common SCM to input parameters, we calculated the Gouy-Chapman Stern (GCS) potential as a function of Stern layer thickness, *d* (which varies due to spatial heterogeneity of a surface) and ionic strength (NaCl concentration) (Fig. 2). A two-fold change in *d* would result in a factor of two difference in the potential drop across the Stern layer as *ΔΦ*_*Stern*_ = *σ/C*, where *σ* is the surface charge density and *C* is the capacitance equal to ε_r_ε_0_/*d* (ε_r_ and ε_0_ are the permittivity of the solution and of the vacuum, respectively). The resulting change in the electric field in the Stern layer (*-dΦ/dz*) would then vary accordingly. Opening the expression for the Stern layer potential drop to allow for spatial variations in all three parameters *(σ, ε*_*r*_, and *d*) will result in variations of the potential across the electric double layer (EDL). It is reasonable to expect that each of these parameters varies by up to a factor of two (for the surface charge density and Stern layer thickness) and by ten (for the relative permittivity). A sensitivity analysis of the ionic strength dependent Gouy-Chapman Stern potential in terms of physically feasible variations in charge density and Stern layer relative permittivity shows that variations of serval hundred mV occur, owing, for instance, to doubling the charge density and halving the Stern layer permittivity (Fig. 2). In contrast, doubling both parameters results in only minor potential differences (Fig. 2). We conclude that expected spatiotemporal variations in the surface charge density and the Stern layer relative permittivity will result in spatiotemporal variations in the surface potential, and the associated electric field, in the range up to several hundred mV. This simple example is directly applicable to other important parameters, including the Stern layer thickness, in mean field or surface complexation models, as alluded to above, and further justifies the proposed probabilistic approach to continuum-scale modeling.

Furthermore, conventional SCMs describe surface properties and reactivities with a single surface acidity constant and surface complexation constant for a given surface and adsorbate (the more advanced SCMs may go as far as to incorporate two- or three-site models with distinct acidity and/or complexation constants). However, new experiments consistently show that nominally similar surface sites (e.g., Si-OH, see ref. ^7^) have vastly different reactivities, which are defined by multiple factors: surface structure, hydrogen bonding in adjacent solution, the surface neighbor species, and aqueous composition. Because continuum-scale simulations rely on empirically fit coefficients to approximate parameter values, they often do not capture experimentally measured outcomes. As we will show below, the mismatch in predicted *vs*. experimentally determined parameters can span orders of magnitude.

The other types of continuum-scale reactive models, which are often utilized in important applications such as nuclear waste storage, are reactive transport models (RTMs) that couple transport equations with chemical reactions, including equilibrium constants and kinetic rate laws. Similar to SCMs, equilibrium constants used in RTMs do not fully reflect the reality of a solid–water interface, where isolated surface sites can have dramatically different reactivities. Furthermore, to model the dissolution of solid phases in RTMs average rates or rate constants are selected^8,9^, whereas experimental evidence indicates that the effective dissolution rate consists of contributions from specific surface sites, where the rates are vastly different^10^. Because surface structure is dynamic, rates may also vary with time^11^, with reaction Gibbs free energy^12^, and with flow rate^13^. Accordingly, reaction rates may vary several-fold for the same crystalline solid, depending on the molecular, crystallographic, and topographic details of their surfaces that change dynamically in time. Therefore, reaction rates are best described by distributions of possible/probable values and not by a singular discrete number.

This Perspective argues that, in place of ensemble averaged constants as input parameters, *probability distributions* are needed to formalize chemical phenomena at interfaces to reflect their heterogeneous nature in SCMs, RTMs, and other continuum-scale models. Current state-of-the-art modeling approaches apply homogeneous chemistry concepts to heterogeneous systems, limiting their applicability and predictive power. A probabilistic approach that captures the stochastic nature of surface sites offers a path forward to bridge detailed molecular-scale information with the continuum-scale models of complex systems. We will show that using probability distributions is appropriate for representing the “surface landscape”, (i.e., the stoichiometry of surface sites, surface charge distributions, and surface topologies), as well as equilibrium constant values and reaction rates. This approach provides a new paradigm that we hypothesize will create a more robust predictive power in continuum-scale models by capturing the wealth of molecular-scale information that is available for interfacial systems. Using molecular-scale information in continuum-scale simulations will advance our capability to model environmental fate and transport, soil system evolution, and to elevate the design and optimization of electrochemical and catalytic processes, desalination membranes, and carbon- and ion-selective capture materials. Achieving this probabilistic approach requires not just advancements in the capabilities of SCMs and RTMs, but also the continued efforts of experimentalists and computational chemists to elucidate molecular details and reactivities of solid−water interfaces.

## Molecular details matter

In this section we will illustrate that a surface is not one reactant but instead a combination of different reactants that are distinct, interdependent, and changing. Recent scientific advances have led to molecular descriptions of interfaces of specific solid–water systems that challenge traditional mean-field models of charged surfaces (see Bañuelos et al. for a comprehensive review)^7^. These studies highlight that molecular details matter as surfaces are heterogeneous at the molecular-scale and cannot be conceptualized as a single “reactant” in interfacial chemistry descriptions. The selected advances illustrated here have been facilitated by new capabilities in scanning probe, synchrotron-based X-ray, and nonlinear optical techniques that reveal the different detailed aspects of the interface under in situ conditions in real-time. Furthermore, computational simulations using density functional theory (DFT) and ab initio and classical molecular dynamics (MD) have been critical in uncovering reaction mechanisms at solid–water interfaces, helping to interpret experimental observables and distinguish the reactivities of different surface sites. These studies have shown that surface sites can have stark differences in their reactivities, such as acidity and surface complexation reactions. Importantly, the surface site reactivity also depends on the local environment, i.e., the reactivity of the same surface site differs depending on the structure and identity of its immediate neighbors.

In the last decade, nonlinear optical methods have greatly enhanced our ability to garner molecular information on buried interfaces, i.e., those surfaces under aqueous solutions. Phase-sensitive measurements have yielded complex spectra generated at solid–water interfaces resolving the orientation of the molecules that contribute to the measured response^14,15^. Moreover, theoretical frameworks used to interpret these measurements now separate the contributions from different regions of the interfacial solution layers and assign them to molecules immediately at the buried surface and those at a distance that are still structurally distinct from molecules in the bulk aqueous phase (the diffuse layer)^15,16^. These methods and related approaches have uncovered the details of hydrogen-bonding networks of water immediately adjacent to a surface (in the Stern layer) and how they are perturbed by changes in pH^17^ and the addition of aqueous ions^18^. Phase-sensitive measurements have allowed also for the total potential to be quantified directly at the surface^19^. This new capability is important, because the surface potential (*ψ*) is one of those approximated quantities that must be incorporated into SCMs (Fig. 1). This quantity is often calculated from mean-field models and rarely had been measured experimentally. Now, the surface potential, which differs from the more commonly measured zeta potential, can be ascertained optically, and at arbitrary ionic strength, using heterodyned second harmonic generation (SHG)^19^ as well as synchrotron-based X-ray photoelectron spectroscopy (XPS) albeit under more limited conditions^20^. This ability to measure the total surface potential provides an important experimental benchmark for the widely used mean-field models for calculating surface potentials and applying the electrokinetic methods used to quantify interfacial potentials. Ultimately, our ability to assess the electrostatics at the surface without having to invoke classic mean-field models, which often rely on semi-empirical parameters and primitive ion models that were put forth based on less sensitive techniques decades ago, will be critical to develop the next generation of surface models and extend them into SCMs and RTMs.

Advanced techniques for measuring *ψ* at oxide surfaces still provide an average value for a given interface. However, we know that charged sites on oxide surfaces are localized resulting from protonation and deprotonation of surface hydroxyls. Whether charges are localized or delocalized significantly impacts both the ion distribution and the net water orientation in the interfacial region according to simulations of charged solid–water interfaces^21^. Specifically, charge localization results in ion accumulation at an interface and local re-orientation of water molecules at interfaces compared with the delocalized charged aqueous interface. Furthermore, recent work using Stark spectroscopy indicates that the local fields can vary significantly across the solid–water interface and that interfacial molecules “sample” this heterogeneous, dynamic environment^22^.

The interfacial charge structure can be changed drastically by high salinity. Lee et al.^23^ observed the salinity-dependent electric double layer (EDL) structure evolution in RbI or RbCl with negatively charged mica surfaces using element-specific resonant anomalous X-ray reflectivity. They found that cations and anions formed alternating discrete layers, causing nonclassical charge overscreening (also referred to as charge reversal) at high salinity. At the silica surface, the impact of overscreening induced by divalent ions with increasing pH on both the water structure and ion speciation within the EDL was also recently observed by Rashwan et al. using vibrational SFG (vSFG) and streaming current measurements^24^.

**Fig. 3:**
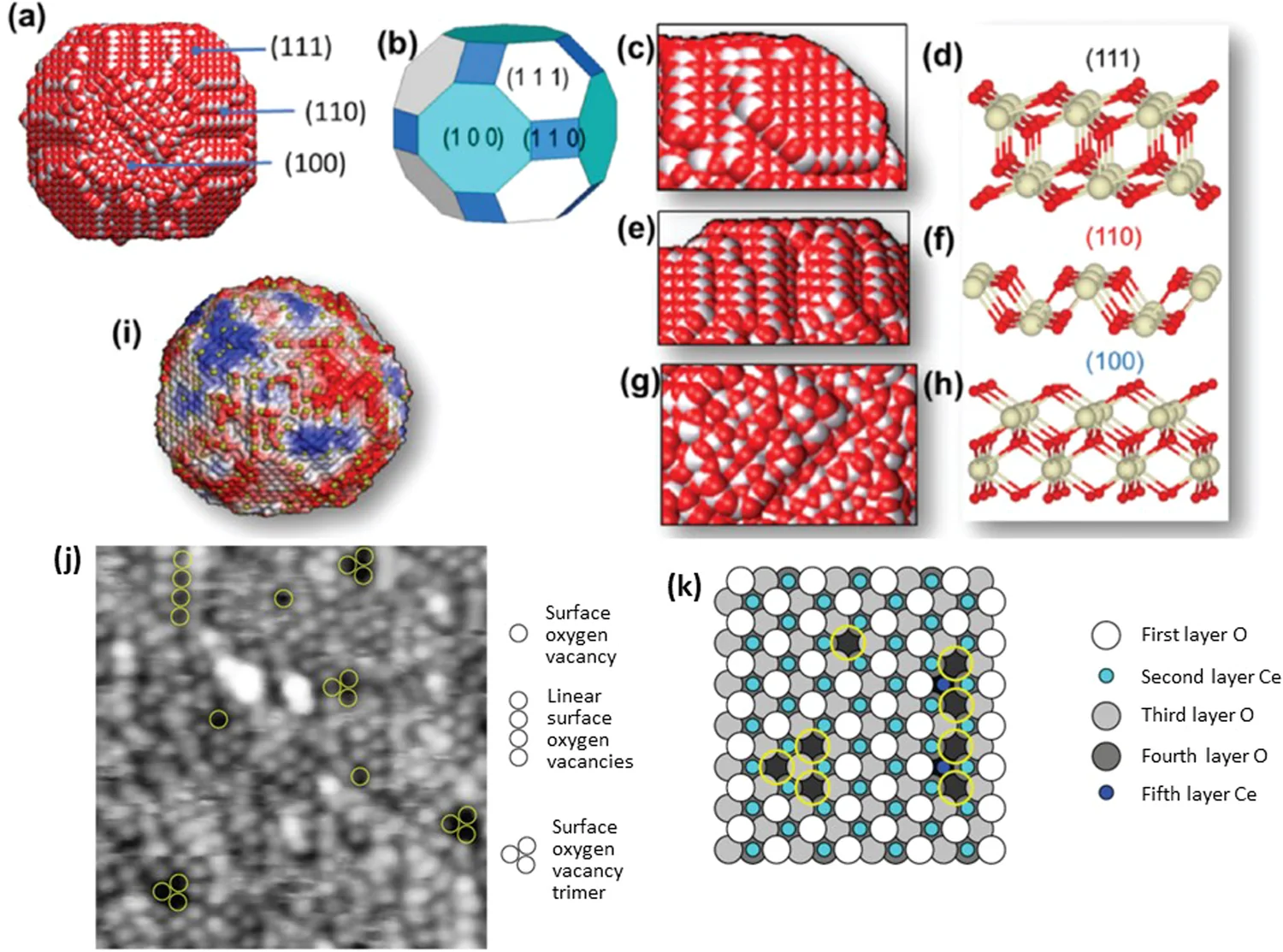
Surface structures of ceria (CeO_2_) nanoparticles. **a** Full atom level model of CeO_2_ nanoparticle; **b** Schematic of CeO_2_ nanoparticle showing crystallographic surfaces; **c** Enlarged view of the CeO_2_ (111) surface showing the presence of surface steps and corners; **d** Perfect (111) surface of CeO_2_ crystal; **e** Nanostructured (110) surface; **f** Perfect (110) surface; **g** Nanostructured (100) surface; **h** perfect (100) surface. Ce = white, O = red; **i** Visualization of catalytic activity of a CeO_2_ nanoparticle surface, where oxygen atoms are colored by their lability—the energetic cost of their removal from the surface. Red-white-blue gradient scale, where red corresponds to labile oxygen (energetically easy to extract) and blue corresponds to oxygen ions that are difficult to extract. The yellow spheres are Ce^3+^ species; **j** Scanning tunneling microscopy image of CeO_2_ surface, a 6 by 6 nm^2^ square is shown from reference^105^; **k** corresponding structural model. Adapted with permission from ref. ^2^ and ref. ^105^.

Experimental methods capable of mapping out the local structure with molecular-scale resolution are transformative tools for characterizing the chemistry of solid–water interfaces^7^. Scanning probe measurements over nearly atomically flat surfaces, such as mica^25^, paired with finite-element analysis^26^ have yielded topographic information on the molecular-scale of both the interfacial potential and water structure. Such methods have been extended to mapping of organic molecules deposited on metal surfaces^27^. Charge profiling three-dimensional (3D) atomic force microscopy has revealed charge layering of ionic liquids on electrodes at Ångstrom depth resolution^28^. 3D fast-force mapping can also estimate the position of individual water molecules in the Stern layer although this emerging method is complicated by data convolution concerns related to tip-specific effects^29^. Other imaging methods such as transmission electron microscopy (TEM), including scanning (STEM), high-resolution (HRTEM), and liquid cell (in situ TEM), in combination with electron energy loss spectroscopy can directly quantify surface structures in dry, humid, or aqueous conditions. Because these measurements are spatially resolved and have near-molecular-scale resolution, they can map out the variety of reactive surface sites on oxide surfaces allowing the abundance of a certain type of surface site to be linked to observable macroscopic reactivities. A well-studied example of this phenomenon is the uptake and release of O_2_ by ceria (CeO_2_) nanoparticles that are widely used in catalysis and other applications. Combined TEM and modeling studies for CeO_2_ have shown that the energetics of O_2_ uptake/release is controlled by (1) specific facets (crystallographic orientation), (2) oxygen site vacancies produced during Ce^3+^/Ce^4+^ redox reaction, and (3) surface hydration (Fig. 3, from ref. ^30^, and ref. ^2^). Because surface defects often produce high energy reactive sites, the emerging research field of defect engineering for nanomaterials is critically tied to these new high-resolution measurements.

Inevitably, the observed heterogeneities of the surface structures discussed above lead to variability in surface properties, such as interfacial potentials, acidities of surface groups (p*K*_*a*_ values)^31–33^, dissolution rates^10^, surface speciation, ion jamming with observed hysteresis in surface acid-base chemistry^33,34^, and heterogeneous nucleation patterns across a single surface^35,36^ Further complicating the situation is the recognition that the surfaces of some materials, e.g., SiO_2_, can have *localized* hydrophilic and hydrophobic regions^36–38^ that have been proposed to produce different surface acidity constants^39^ in concert with changes in hydrogen bonding effects on the distribution of silanol site acidities^40^.

**Fig. 4:**
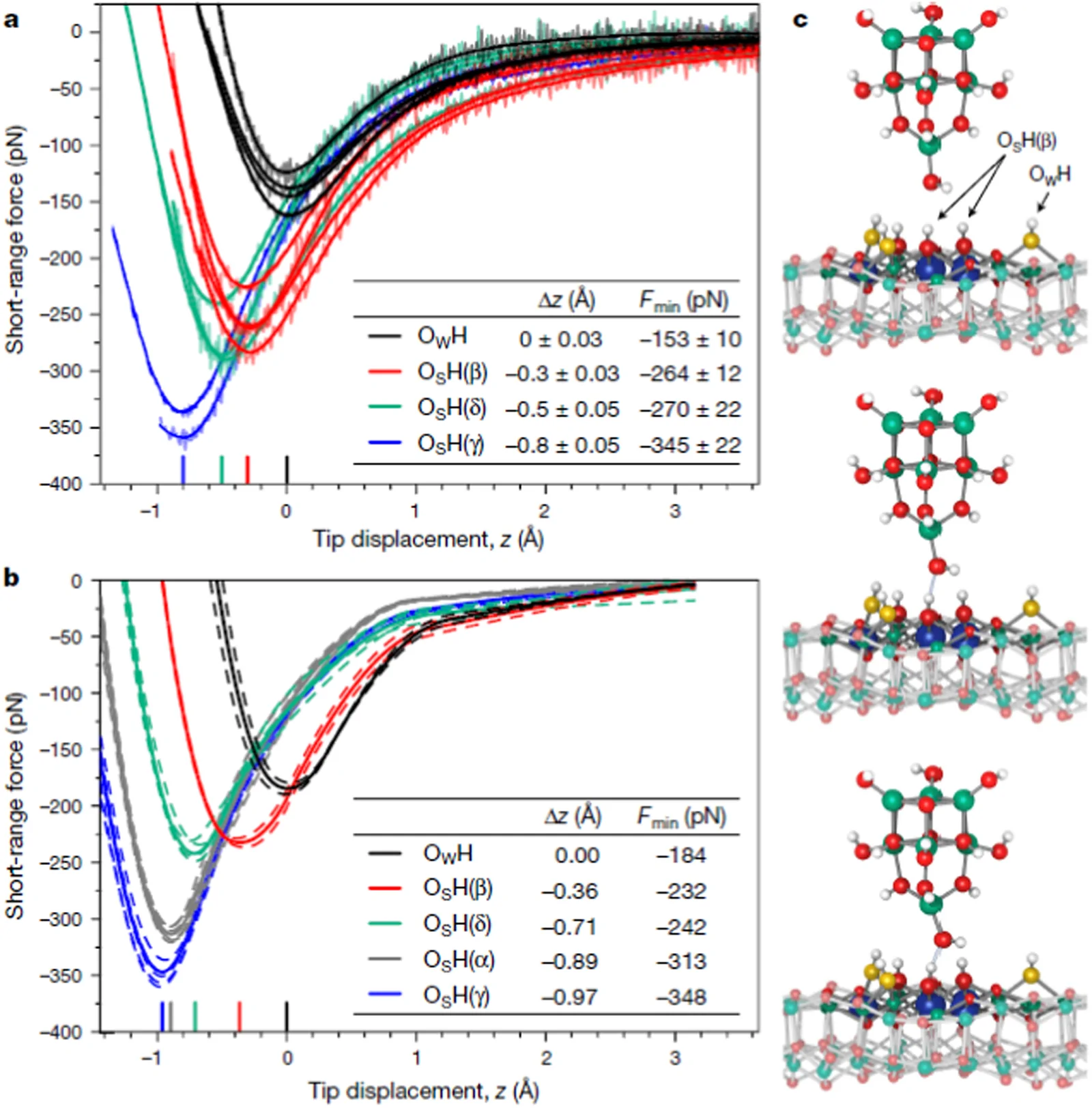
Probing individual hydroxyls on In_2_O_3_ surface with an Atomic Force Microscopy tip. **a** Experimental short-range force–distance curves for the OH groups; **b** Calculated short-range force–distance curves for the OH groups; **c** Tip–sample configuration for various tip-surface separations. O_W_, yellow; H, white; O, red; In, blue and green. Reprinted from reference^41^ with permission from Springer Nature, © Wagner et al. under exclusive license to Springer Nature Limited 2021.

Capturing the acidity of surface groups is of specific interest to SCM and RTM development because site charge influences surface reactivity and may vary greatly on the same surface^41–43^. A recent significant and surprising finding by ref. ^41^. who combined non-contact AFM measurements and DFT modeling indicates that surface hydroxyl groups at an In_2_O_3_ (111) surface have p*K*_*a*_ values varying several orders of magnitude, based on the H-bond strength measurements at individual surface sites (Fig. 4). Multiple distinct p*K*_*a*_ values have also been observed for silica in both theory and experiment under aqueous conditions^31,39,40^. Therefore, the relative abundance of different sites varies significantly, which we propose should be represented as a probability curve in continuum models.

Although imaging surface structures, localized surface potentials, local p*K*_*a*_ values, and particle–particle interactions are paramount to understanding these systems, it is equally crucial to capture time-dependent fluctuations referred to as surface dynamics. Most environmental interfaces are intrinsically dynamic and sensitive to changes in pH and the presence of ions as they consist of amphoteric sites that become charged and interact, either in a specific or non-specific manner. Generally, models consider that a given solid will exhibit a trend in affinity towards ions based on its composition. Yet, recent experimental work for the planar^44,45^ and nanopore silica–water interfaces^46^ reveals that such trends in ion affinity can be significantly altered as the pH is changed. One hypothesis that can qualitatively explain a change in relative ion affinity is that the ions can interact with at least two distinct sites on the silica surface, one charged and one neutral, and as the relative ratio of charged to neutral sites increases with pH so does the affinity for ions in solution^45^. Current work aims to investigate whether revising SCMs to include two-site binding of cations can capture such pH-dependent trends in ion affinities. Furthermore, changes in pH, ion concentrations, and solid chemistries might reveal that a probabilistic approach capturing distributions of affinities, rather than two affinity constants, can better predict such behavior.

Real-world solid–water interfaces must also contend with dynamic chemical and geometric complexities: the composition of the aqueous phase at the solid–water interface is multi-component where competitive adsorption plays an important role in Stern and diffuse layer structures^47^. Continuum-scale models must capture the dynamics and coupled behavior between adsorbates, water, and surface site structures. Furthermore, nanoconfinement of surfaces often leads to anomalous chemistry where interfacial reactivity is dictated by the spatial dimension of the reactive solid–water interface^42,48,49^. In particular, in nanopores, the polarization force between ions and the solid surface at an interface determines ion propensity toward nanoconfined spaces^49^.

Capturing interfacial reactivity is further complicated by the fact that the speciation of adsorbed ions, and likely of surface sites^50^, can vary with ionic strength and surface coverage. For instance, several classic linear and nonlinear optical measurements as well as atomistic simulations have shown that surfaces functionalized with carboxylic acids remain neutral (uncharged) even at highly basic pH values^51–53^. The underlying mechanism is one in which Coulomb repulsion within the surface plane is largely reduced when the carboxylate groups pick up a proton from the aqueous solution to form carboxylic acid dimers, similar to those found in glacial acidic acid (an insulating liquid). A similar phenomenon might be occurring for bare oxides such as silica where a bimodal distribution of acidities has been observed for silanol sites above the point-of-zero-charge^31–33^. Likewise, Sr^2+^ as well as some lanthanide cations (nominally 3+ in solution) exist as singly-charged species when they are absorbed to certain surfaces^54,55^. In this case, the underlying mechanism likely involves replacing a water molecule from the ion’s hydration sphere with a counter-ion, such as chloride or surface deprotonation to create an OH^-^ group^56,57^. Sr^2+^ then absorbs as the [SrCl]^+^ ion pair, which is subject to reduced lateral Coulomb repulsion. SCMs should take this effect into account, but currently do not. This is a problem of exponential sensitivity, as the Boltzmann term governing the surface coverage is raised to the power of the charge of the adsorbed ion. If this charge changes from 3 to 2 or from 2 to 1, the exponential sensitivity indicates a much different surface coverage relative to what is expected from bulk thermodynamics. We note that such ion-pairing effects are routinely observed in brine solutions (NaCl > 1 M)^58^, but they occur at surfaces when electrolyte concentrations are orders of magnitude smaller compared to brines, for example at the fused silica/water interface Sr^2+^–Cl^-^ ion pairing occurs once NaCl concentration reaches only 10 mM. The surface-promoted ion-pairing processes need to be incorporated into new descriptions of Stern layer for ion speciation. When RTMs solve for chemical speciation, they do not incorporate surface-promoted shifts in speciation as described here.

The presence of salts (electrolytes) can influence solid–water interactions, including surface complexation, dissolution, and precipitation reactions. Since the work of Dove^59^ and coworkers on silica dissolution, researchers have attempted to further unravel the details of salt effects on solid–water interfaces. For example, Icenhower and Dove^60^ found that dissolution rates can increase by over 20 times in 0.05 M NaCl solution compared to de-ionized water. Notably, the same experiments show that the activation energy (74.5 ± 1.4 kJ mol^−1^) in the range of 25 to 250 °C does not change within experimental error with this increase in rate constant. This suggests that the Arrhenius pre-exponential factor (A) related to the activation entropy of the reaction is changing rather than the activation enthalpy. Kubicki et al.^61^ hypothesized, based on DFT-MD simulations, that the dissolution entropy is made more favorable when salts are present at the interface due to changing H-bonding that favors intra-surface H-bonds and thus H^+^-transfer and hydrolysis of Si–O–Si linkages leading to dissolution. This observation of H-bonding changes is consistent with vSFG experiments by ref. ^18^, revealing the decrease in ordered water in the Stern layer at the silica surface upon salt addition. Likewise, ref. ^62^. showed that salt impacts structured interfacial water most significantly near neutral pH where the effect of salt on accelerating silica dissociation is greatest^59,60^. Other simulations and time-resolved vSFG (TR-vSFG) spectroscopy have found similar behavior with addition of salts^63,64^.

**Fig. 5:**
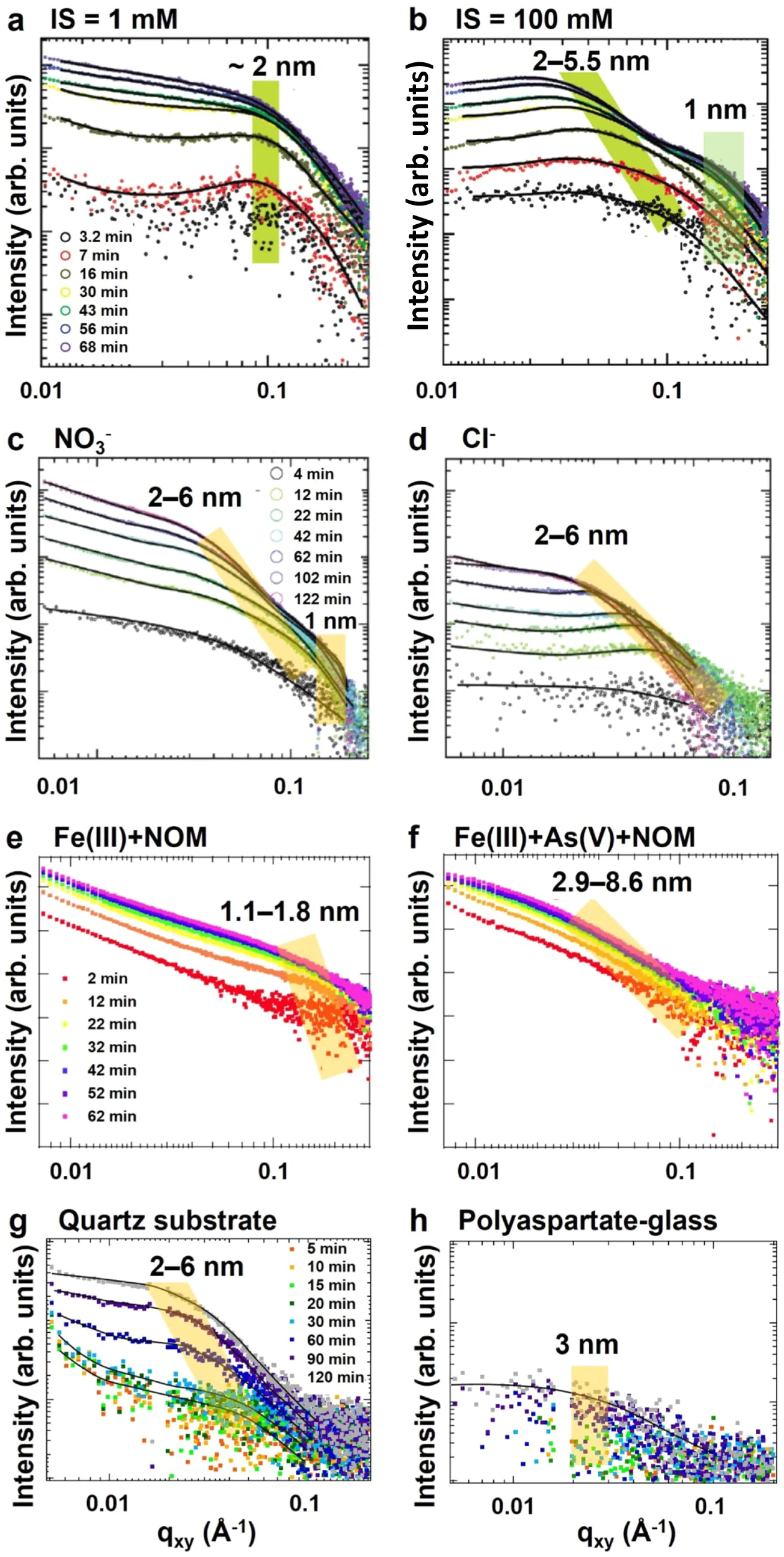
In situ observations of the nucleation and growth of iron (hydr)oxide nanoparticles in varied aqueous environments with small angle X-ray scattering. In situ measurements of heterogeneous nucleation on quartz substrates in a solution containing 10^−4^ M Fe(NO_3_)_3_ at pH 3.6 ± 0.2 by grazing incidence small angle X-ray scattering (GISAXS), showing in-plane (*q*_*xy*_) 1D scattering. The shaded boxes indicate the particle size evolution with reaction time. Adapted with permission from ref. ^36^ (**a**) With 1 mM NaNO_3_ ionic strength (IS), nucleation is dominant. **b** With 100 mM NaNO_3_ IS, particles grew from ~2 to 5.5 nm, with the formation of secondary ~1 nm particles. Detailed discussion about images (**a**, **b**) is available in ref. ^66^ (**c**) With 10 mM NaNO_3_, both nucleation and growth were observed. **d** With 10 mM NaCl, although the particle size is comparable to the nitrate system, the total particle volume does not increase, indicating Ostwald ripening. Detailed discussion about images (**c**, **d**) can be found in ref. ^106^ (**e**) In the presence of natural organic matter (NOM), particles aggregate, as indicated by power law scattering at low *q*. **f** In the presence of both arsenate and NOM, large particles are also observed. Further discussion about images (**e**, **f**) is available in ref. ^67^. **g**, **h** The influence of substrate chemistry is evaluated by coating the surface with hydrophobic polyaspartate. More information about images (**g**, **h**) can be found in ref. ^68^.

In addition to dissolution, the salt concentrations and types can affect the nucleation of metal (hydr)oxides and their subsequent growth and Ostwald ripening. For example, Li and Jun examined the effect of salinity on CaCO_3_ nucleation on quartz using grazing incidence small angle X-ray scattering^65^. When salinity increased from 0.15 to 0.85 M NaCl, effective interfacial energies dropped from 47.1 mJ/m^2^ to 36.4 mJ/m^2^, thus decreasing the thermodynamic penalty of nucleation. However, the kinetic factor for nucleation (*J*_*0*_)—related to ion diffusion and nuclei surface properties—reduced ~13 times. Lower *J*_*0*_ values resulted from slower CaCO_3_ monomers impingement rate caused by decreased electrostatic attraction at high salinity, which is also consistent with charge overscreening at high salinity. Based on these thermodynamic and kinetic contributions to the CaCO_3_ nucleation, the net nucleation rates could increase an order of magnitude at higher salinities. Furthermore, as shown in Fig. 5, the nucleation and growth of iron (hydr)oxide nanoparticles are also controlled by many aqueous solution variables, such as the salinity^66^, types of salt ions, co-existing oxyanions^67^, and natural organic matter^68^. Even with this known complexity, RTMs typically consider solid nucleation process to be instantaneous or start as soon as solution reaches the saturation index for a given phase, and do not count the nucleation step as a discrete part of the process. This oversimplification of nucleation processes can result in discrepancies between experimental findings and RTM results^69^.

As shown above, the chemical complexity of even simple oxide–water interfaces is daunting from a molecular perspective. These surfaces become even more complicated in the presence of organic and microbial communities. Grassian and co-workers have shown that dissolved organic matter can coat oxide surfaces at low and circumneutral pH^70–74^. Moreover, surface adsorption from complex aqueous phase systems containing biomolecules, humic, and fulvic substances show that larger complex macromolecules adsorb onto mineral surfaces in a manner that depends on solution pH and ionic strength. Similarly, biological components such as proteins adhere to oxide surfaces to form an “eco-corona”^75^ and the protein-oxide surface interactions depend on pH, the nature of the surface, and neighboring oxyanions^70–72^. Environmental DNA (eDNA) can attach to oxide particle surfaces, but little is known about these interactions and how they impact the underlying surface structure and reactivity as well as the stability of adsorbed eDNA^76^.

These cumulative findings further support two notions: (1) interfacial water structure is a key player in interfacial reactivity and (2) salt ions are not spectator species at solid–water interfaces. We have shown how surfaces are heterogeneous on all scales of interest, and how interactions with complex molecular species that are typically present in the environment make these systems hard to study. Additionally, in low humidity environments, such as Earth’s atmosphere, the surface heterogeneity of single particles can control water adsorption on surfaces as a function of relative humidity^7,77^. Specifically, edge and defect sites adsorb water preferentially from the gas phase as a function of increasing relative humidity prior to the adsorption of water on planar surfaces. The spatially resolved studies, including infrared nanospectroscopy^77^, show how surfaces are heterogeneous and water does not uniformly coat the surface, meaning that only select surface sites can participate in reactions.

A challenge, as well as an opportunity, moving forward is to utilize the state-of-the-art tools to examine more realistic, chemically/structurally heterogeneous surfaces in complex environments that contain ions, dissolved organic matter, and biological components to understand main molecular controls on surface reactivities. We can then test the hypothesis proposed here that describing main reactivity parameters with probability curves leads to more accurate continuum-scale models. Le Traon et al.^78^ highlights that reaction kinetics in porous systems deviates from the batch experiments by orders of magnitude, demanding that experiments and simulations more realistically capture larger scale effects. This possibility raises several thought-provoking questions such as: *Do aqueous and solid phase complexities produce a heterogeneous surface with different domains? Are the surfaces “patchy” with some hydrophilic and hydrophobic domains, and some regions enriched with adsorbed species (or covered with organic matter)? Can these complex surfaces be described by probabilistic models to capture all types of reactive surface sites for all surface domains?* These are difficult yet important questions to resolve to understand the full chemical complexity of solid–water interfaces in the environment.

In the following section “Rectifying the Molecular View with Ensemble Models” we will discuss examples where interfacial processes were successfully incorporated into continuum-scale models, as well as those cases where such models cannot be constructed without a complete re-working of the mathematical and statistical approaches on which they are built.

## Rectifying the molecular view with ensemble models

**Fig. 6:**
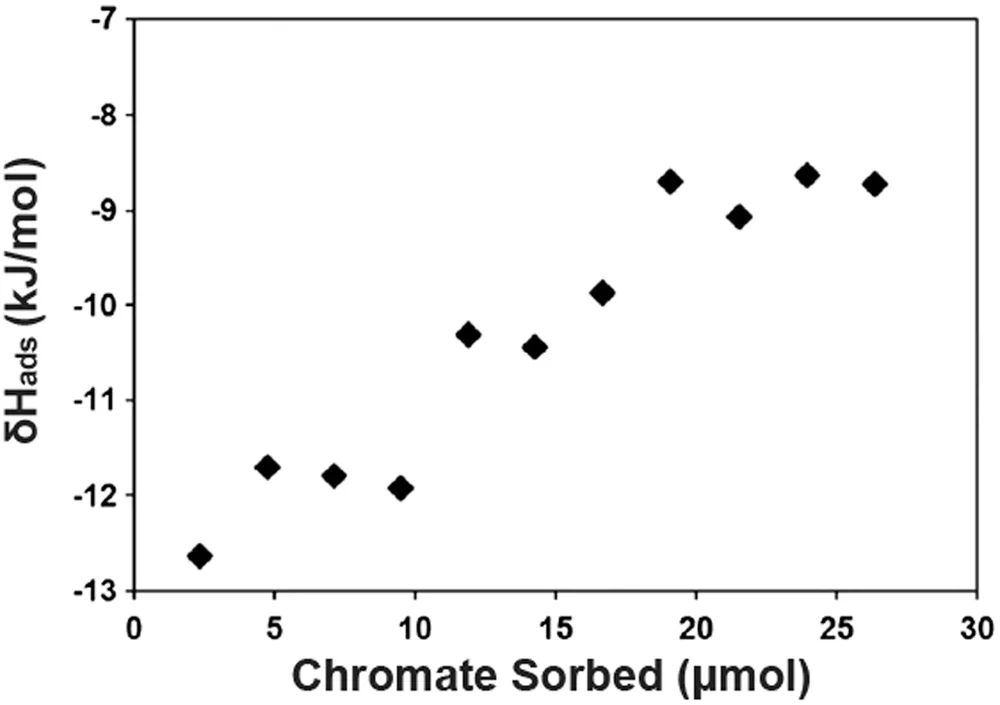
Heat of adsorption measurements using operando flow microcalorimetry. Differential molar enthalpies (δH_ads_) measured by *operando* flow microcalorimetry for the sorption of chromate on ferrihydrite, showing that the values become less negative (less favorable) with increasing surface loading. Adapted with permission from ref. ^107^.

In this section we will show how mean-field models work in some instances but not in others. To take the heterogeneity of reactive sites during adsorption into account, a commonly used equilibrium adsorption model at a solid–water interface is the Freundlich isotherm, which theoretically accounts for heterogeneous surface sites. Yet only one affinity constant describing bonding strength is derived from adsorption data and this averages the enthalpy of adsorption *ΔH*_*ads*_ for all sites. If the range of *ΔH*_*ads*_ is narrow, using one constant value would not be a major issue; however, inverse adsorption chromatography^79^, and *operando* flow microcalorimetry have demonstrated that the range of *ΔH*_*ads*_ values for the same sorbent–sorbate (surface–ion) pair can be up to 200 kJ mol^−1^! Thus, one can infer that the variation in *ΔH*_*ads*_ is not a simple matter of adsorption reactions at the same type of sites, which is less favorable with increasing sorbate coverage (Fig. 6). Instead, the *ΔH*_*ads*_ variation reflects different types of surface sites with distinct bonding mechanisms, consistent with the notions of local spatial heterogeneity and stochastic distribution of surface reactivities discussed earlier. Many adsorption isotherm studies report better fits to the data at the mid-range of solution concentrations and are less accurate at the low- and high-concentration tails^80^, which is indicative that the values at the higher and lower tails of the probability curve are ignored. Because surface defects likely have the most negative *ΔH*_*ads*_ values and lowest surface site densities (representing tail ends of the site probability curve), they have not been modeled accurately. Considering that in many real-world scenarios, the sorbates are present at trace levels, the applicability of models based on ideal surfaces at higher aqueous concentrations that are typically studied in a laboratory setting becomes questionable. There are also critical needs for thermodynamic data and computational chemistry models that can address the lower concentrations and reactions at surface defects^81^ and in nanopores^46,49^ to obtain predictable thermodynamics and kinetics under realistic environmental conditions.

One promising example is the determination of Fe-oxy(hydr)oxide reactive sites that has been translated into a SCM capable of unifying adsorption equilibrium constants for the important contaminant chromate^82^. Bompoti et al.^82^ utilized the MUSE algorithm and found that in SCMs it was easiest to keep the reactive site densities fixed for each solid and vary the solid concentration and capacitance until the model agreed with experimental data. High resolution data, for example using STEM HAADF helps determine the crystal face contributions for different surface sites, and the respective site densities characteristic for each surface that can be incorporated in SCMs^83^.

**Fig. 7:**
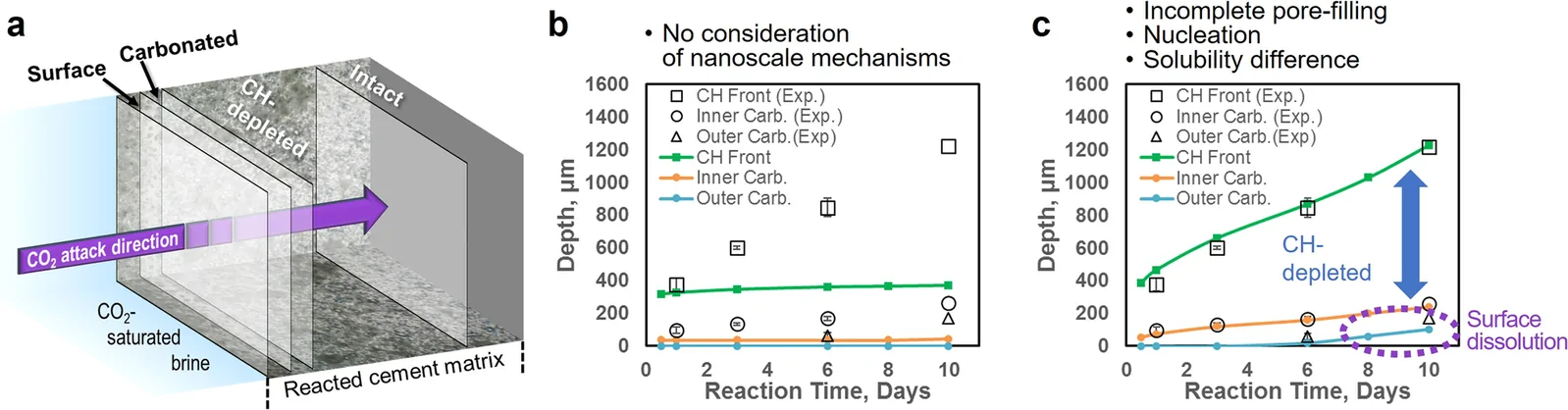
Incorporation of nanoscale interfacial reactions into a reactive transport model. **a** Illustration of direction of CO_2_ attack into the cement matrix. The cement samples were reacted in a CO_2_-saturated brine (0.5 M NaCl) with a solid-to-liquid volumetric ratio of 1/16. The solution was equilibrated at 95 ^o^C under 100 ± 5 bar of CO_2_. A total alteration thickness of 1220 ± 90 μm was observed, including a 960 μm CH (Ca(OH)_2_, portlandite)-depleted zone, a 100 μm carbonated layer, and a 170 μm surface region. Interfaces between zones are drawn to scale. **b**, **c** Modeling results with and without sufficient consideration of nanoscale mechanisms in comparison with experimental data. **b** Results with no consideration of nanoscale mechanisms. **c** Results with consideration of incomplete filling of pore space at nanoscale, nucleation kinetics, an enhanced solubility in confined pores. By incorporating nanoscale evolution of interfacial chemistry into RTM can generate a better match with experimental observations. Adapted with permission from American Chemical Society from ref. ^69^.

When considering larger scales in RTMs, the dynamic evolution of solid–water interfaces can significantly alter the fate and transport of ions, which is not fully captured in current models. Adsorption of chemical species and temporal evolution of solid phases due to dissolution-precipitation processes changes reactive site densities and types. Until recently, RTM could not include solid nucleation due to the lack of experimental information about nucleation. Instead, it captures precipitation as a group term by assuming that nucleation is instantaneous, and only includes the solid’s growth rate. RTMs also do not capture pore-size effects on solubility and nucleation kinetics. Recent advances have been made to incorporate experimentally obtained kinetic and thermodynamic information (e.g., nucleation rates, activation energies, and interfacial energies)^65,84,85^ of calcium carbonate nucleation into an RTM code CrunchTope. The incorporation of nanoscale interfacial reactions into the RTM improved the model accuracy of both the evolution of the Ca(OH)_2_-depleted zone and the surface dissolution zone at supercritical CO_2_–brine–cement interfaces (Fig. 7)^69^. Experimentally-obtained nucleation thermodynamic and kinetic information are important in scaling up nanoscale observations of chemical reactions to larger scale predictions. Similarly, this improved RTM framework can be utilized to predict managed aquifer recharge (MAR) where reclaimed water is used to replenish underground reservoirs. The reclaimed water for MAR is rich in dissolved oxygen, which can alter the dissolution of minerals with toxic components such as arsenic-bearing iron sulfides and lead to subsequent iron (hydr)oxide nucleation and toxic species adsorption onto the newly formed iron (hydr)oxides^86–89^. Understanding the nucleation and dynamic interfacial chemical processes and incorporating them into RTMs will significantly improve the predictions of pollutant mobility, benefiting safer aquifer management to address water shortage problems.

## The way forward: towards predicting and controlling interfacial behavior

A logical next step for improving the accuracy of continuum-scale models is to increase the number of discrete parameters used in these models (e.g., use two p*K*_*a*_ values instead of one). Such approaches have already been explored and do indeed show increased accuracy^90^. However, should the splitting of single variables into sets of discrete values (multiple-parameter approach) be the way forward? We argue that it should not be, and a paradigm shift is urgently needed. The fundamental question remains—can we keep applying homogeneous chemistry concepts to heterogeneous systems? The probabilistic nature of chemical phenomena in homogeneous systems has been addressed by statistical mechanics—e.g., the Boltzmann distribution describes the physical nature of molecules in populations having different states, the likelihood of which changes based on the conditions imposed on these populations. Because gaseous or aqueous systems are well-mixed, the Boltzmann distribution is usually Gaussian. When we consider solid surfaces involved in interfacial reactions, a “well-mixed” state is fundamentally impossible for any realistic solid surface. Current molecular models and spatially resolved measurements can capture surface heterogeneity and characterize the localized reactive domains on surfaces at molecular-, nano-, and other scales discussed in “Molecular details matter” section of this Perspective. The problem is that the continuum-scale models, such as SCM and RTMs, are not designed to incorporate spatially differing reactivities of surfaces. We propose that probability distributions of surface descriptors instead of average constant values should be used to formalize interfacial properties in continuum-scale models. Therefore, using probabilities to describe surface properties is a more promising approach in comparison to the stepwise increase in the number of variable values used in multi-parameter sets. Including probability distributions for the variables of interest could result in efficient continuum-scale models because localized effects will be incorporated within non-localized parameterization schemes. Hence, this approach has the potential to address surface heterogeneity at different scales. If successful, this new paradigm will lead to scale-independent, universal models that would allow for the prediction of interfacial reactivities in complex chemical systems for the first time, a dream come true for scientists and engineers in many research fields.

To begin, we need to develop new mathematical frameworks and computational approaches to describe chemical parameters and properties as probability distributions, instead of ensemble average values, to reflect real-world complexity and to generate scaled-up SCMs and RTMs. We propose that accounting for chemical and structural complexity in such new generation SCM and RTM codes requires re-writing them using a fundamentally new approach. As shown in our examples above, reaction rates, equilibrium constants, and surface acidity constants vary across a surface and correlate to distinct structural characteristics (e.g., oxygen vacancies, crystallographic orientation, local structure of amorphous phases, sorbates, and “spectator” ions). We anticipate that normal, bell-shape curves could sufficiently capture the relevant parameter space in some cases where stochastic processes dominate, while in other cases where surface reactivity is a sum of non-random phenomena, they will be best described by more complex types of probability curves. We advocate for applied mathematicians and statisticians to become more involved in interfacial chemistry research to develop rigorous descriptions of interfacial processes for specific use in RTM and SCM codes. The inspiration for such models can be drawn from molecular-scale probabilistic algorithms, including Metropolis Monte Carlo (statistical sampling of energetic states)^91^ and Kinetic Monte Carlo (sampling of reaction rates)^92^. These models are currently limited to molecular-scales. From the experimental side, approaches that can quantify the spatiotemporal variation of heterogeneous rates, adsorption free energies, as well as interfacial capacitance, relative permittivity in the Stern layer, and distribution of electric fields are needed to inform these models.

**Fig. 8:**
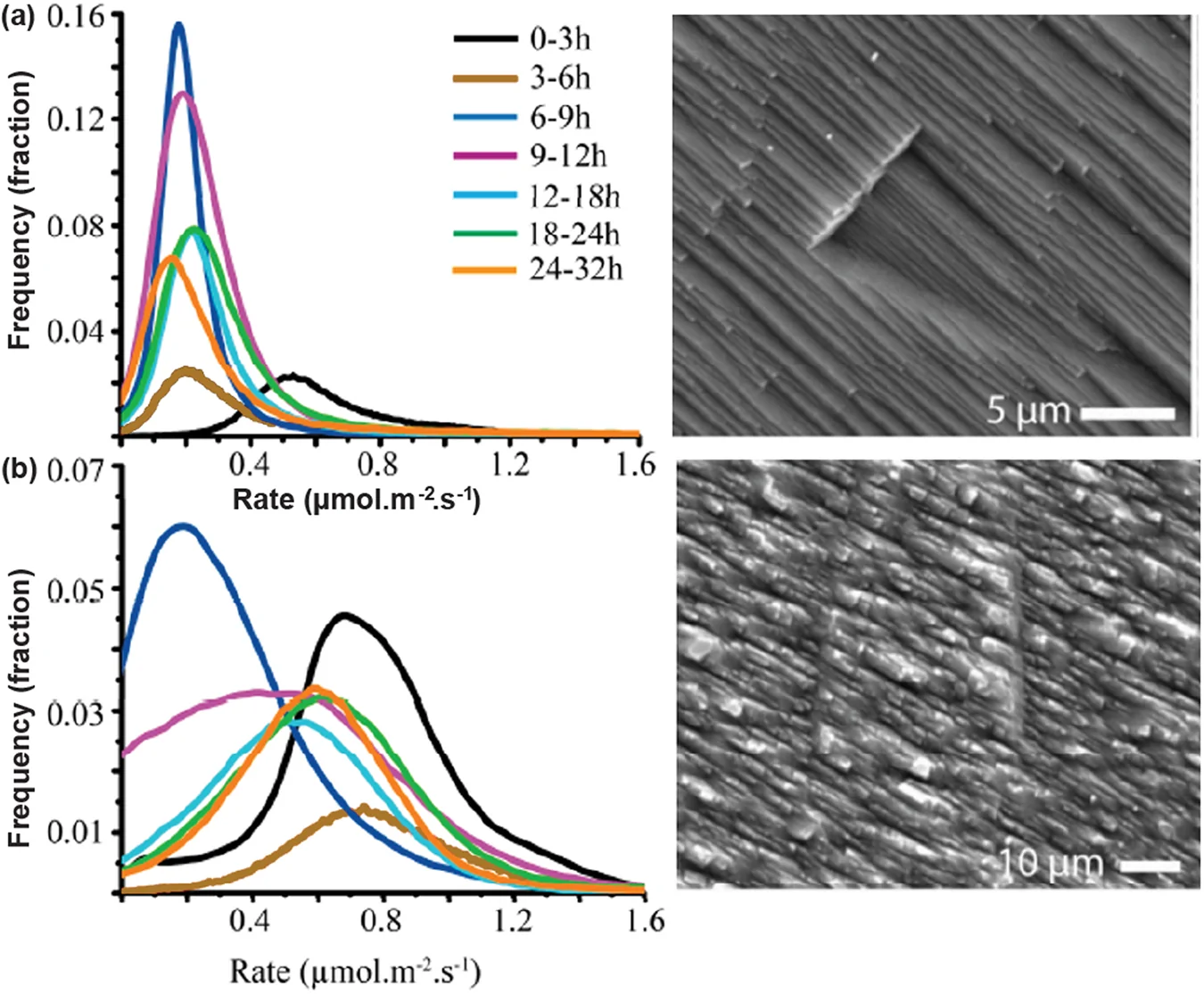
Probability distributions of calcite dissolution rates measured in laboratory dissolution experiments. **a** Dissolution rate spectra for “striated” surface; and **b** Dissolution rate spectra for “hill-and-valley” surface. Adapted with permission from Trindade Pedrosa et al.^95^.

In the geoscience community, Lüttge and co-authors proposed using stochastic models to capture mineral dissolution processes^93–96^. This conceptual approach was motivated by high-resolution in situ measurements on carbonate and silicate surfaces in aqueous solutions. These measurements clearly indicate site-dependency and time-dependency of the dissolution rates, where the probability distributions evolve in time (Fig. 8). For calcite surfaces in Fig. 8, we see that the initial surface topography has a measurable impact on the mean rate values (peaks in the distribution curves) and on the width of the distributions. In fact, a dissolution rate is more accurately represented by a term “rate spectra,” given the variability and gradual changes across a given crystalline surface^10^. Importantly, Lüttge et al. developed an initial framework for treating dissolution phenomena using a probabilistic approach with the dissolution probability defined as^96^:

1 $${P}_{i}={\prod }_{j=1}^{i}{P}_{j}$$

Here, ${P}_{i}$ is the dissolution probability for a molecule with *i* bonds to the surface written as the product of hydrolysis probabilities over all bonds. Furthermore, the logarithm of probability for an individual surface unit to be dissolved is proportional to the sum of activation energies for bond hydrolysis $\varDelta {E}_{{ij}}$^93^:

2 $${{{{{\mathrm{ln}}}}}P}_{i}=-\frac{\mathop{\sum }_{j=i}^{i}\varDelta {E}_{{ij}}}{{kT}}$$

where *k* is the Boltzmann constant, and *T* is temperature. We must note that the variability in the measured dissolution rates shown in Fig. 8 is 2 to 3-fold, because these measurements were conducted on the same crystallographic surface. For numerous solids, the difference in dissolution rates for different crystallographic terminations may reach orders of magnitude. Therefore, for realistic solids the probability weighted approach is crucial, because averaging and ignoring this variability may result in model predictions that are “off” by orders of magnitude.

Guren et al.^97^ illustrate how to derive a set of dissolution rate probabilities from Kinetic Monte Carlo simulations and then how to use them as input into the macroscopic stochastic model. The result of this rigorous procedure is an accurate representation of mineral dissolution that takes place at different surfaces and surface sites of the same material. Regarding RTM, an approach for parameterizing heterogeneity in surface reactivity has been recently demonstrated using nanotopographic images to generate a distribution of surface slope factors that act as a correction factor for the RTM-calculated rates. This approach led to much better agreement between the simulated dissolution rate maps and rate spectra than the standard RTM^98,99^. While these examples are extremely promising and represent an advance in the field of reaction modeling, the results are still limited to simple systems. A major break-through is needed for translating chemical knowledge from molecular-scale into continuum-scale models.

**Fig. 9:**
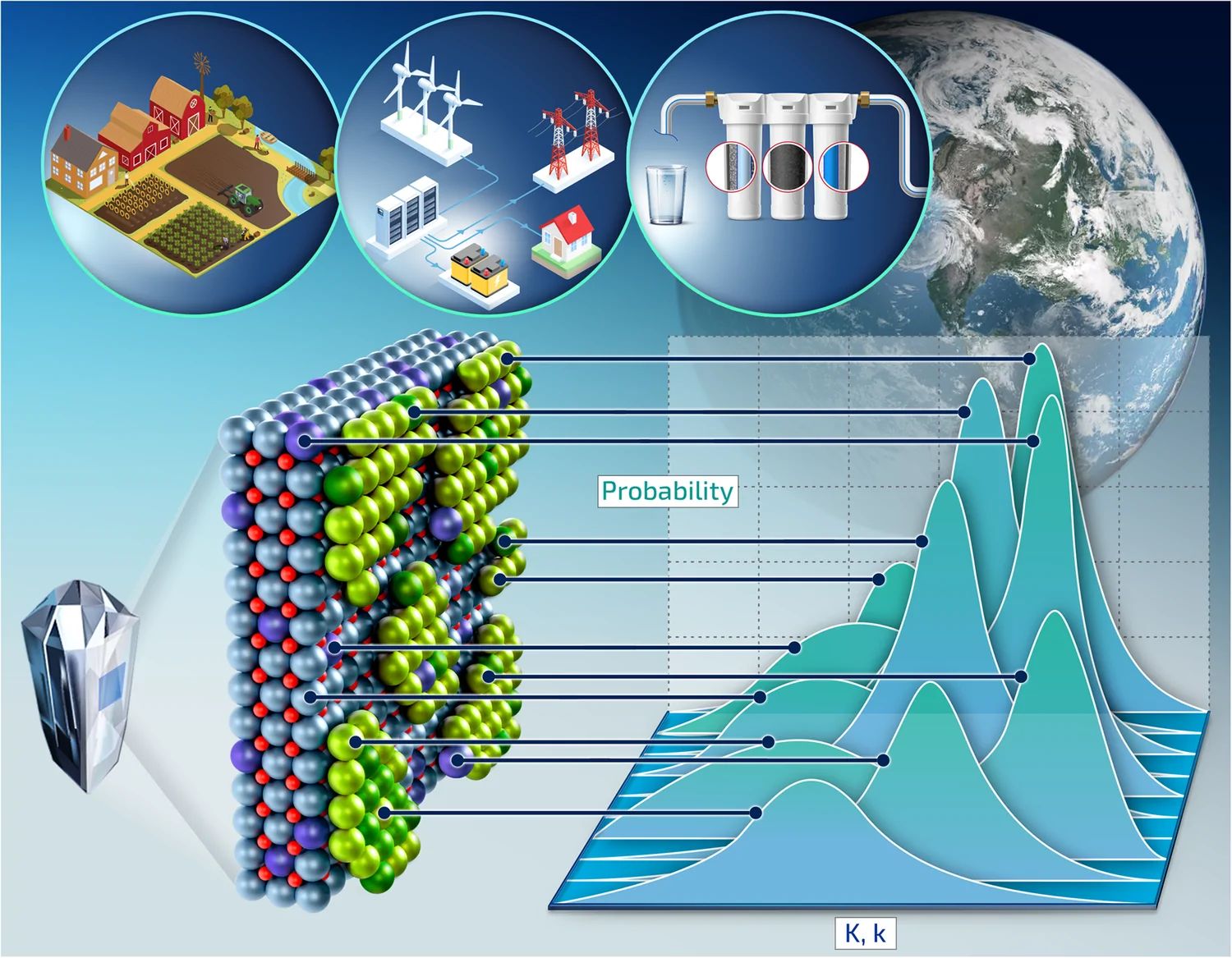
Probabilistic nature of surface sites and their reactivities. Schematic representation of various surface structures on a single crystal surface that have reactivities best represented by a distribution of equilibrium constants (K) and rection rate constants (k). These dynamic surface reactions include solvation, surface complexation, and attachment-detachment processes due to dissolution or precipitation. Chemistry of solid-water interfaces is crucial for understanding environmental fate and transport, and for applications such as water treatment, conventional and unconventional energy, and agriculture (all of which are represented with symbols in the upper portion of the figure).

In this Perspective we propose that an approach that captures probability distributions must be applied in SCMs and RTMs to encompass *all relevant constants and surface properties*, including dissolution rates and nucleation and growth rates, when considering chemistry of solid–water interfaces (Fig. 9).

Developing new methods for incorporating probability distributions into SCM and RTM codes for the numerous reactive surfaces present in the environment will be possible by utilizing new computational approaches. A longstanding grand challenge in computational science has been the seamless transfer of information across scales from molecular to field-scales^100^. In practice, this ideal has not been achieved because funding for multi-scale modeling efforts have not been the norm and computational limits have not allowed significant overlap in spatiotemporal scales among the various approaches. The latter obstacle can be overcome with the advent of exascale computing and the development of codes that incorporate machine learning (ML)-based interatomic potentials or ML-IAPs^101^. Connecting atomistic and pore scale simulations through advanced computational power can be achieved by systematic development of interatomic potentials via machine-learning. Exascale computing makes simulations of 10^7^ atoms over durations of microseconds possible, and the ML techniques allow for the development of accurate, reactive IAPs based on experimental data and quantum results. Thus, it would be possible to perform atomistic simulations that overlap with the mesoscale and can more realistically represent solid–water interfaces. Exascale computers will allow for accurate atomistic simulations of reactions and flow on scales that overlap the micron-scale elemental volumes of lattice Boltzmann simulations^102^. Coarse-grained mesoscale simulations (i.e., mesoscale) allow for larger and longer spatiotemporal scales that overlap finite element and continuum methods. This “bottom-up” approach can provide parameters that are useful in larger scale models such as SCM (e.g., ref. ^103^). Additionally, ML can be used to identify feature importance, value clustering, and detecting anomalous values^104^, all of which can aid in the statistical descriptions of interfacial reactivities. Smaller scale simulations can be used to test assumptions and approximations made for larger scale simulations while simultaneously providing chemical mechanism information that could be incorporated into SCMs or RTMs. By incorporating probability distributions and integrating across scales with experiments and simulations, it will become possible to derive new modeling paradigms that are consistent with field observations and incorporate molecular-level information. This approach will enable bridging of laboratory experiments with modeling efforts to predict chemical transformation in complex industrial systems and natural environments, including critical settings such as nuclear waste sites. Similar approaches can be used for predicting catalyst performance and to design fit-for-purpose materials for energy and the environment. With exponentially rising computational power, the advancement in machine learning and artificial intelligence tools and the increasing spatiotemporal resolution of laboratory measurements, this perspective provides a conceptual framework that could enable sustainable solutions to global problems including clean water, renewable energy, and climate change.
